# Mother–Child Reminiscing About Emotionally Negative Events and Children’s Long-Term Mental Health

**DOI:** 10.3389/fpsyg.2021.632799

**Published:** 2021-10-05

**Authors:** Jessie Bee Kim Koh, Qi Wang

**Affiliations:** ^1^Culture, Self and Emotion Development Lab, Applied Psychology Program, The Chinese University of Hong Kong, Shenzhen, Shenzhen, China; ^2^Culture & Cognition Lab, Department of Human Development, Cornell University, Ithaca, NY, United States

**Keywords:** emotional reminiscing, mental health, culture, mothers, children, American, Chinese

## Abstract

The present study examined the longitudinal relation between mother–child reminiscing of emotionally negative events and children’s mental health. European-American and Chinese-American mothers discussed with their 4.5-year-old children an event that was emotionally negative to the child. At age 7, children’s mental health was assessed, including measures for externalizing problems, internalizing problems, negative social self (an Asian-salient dimension of depression), behavioral problems, and socially adaptive behavior. Independent of culture, maternal reference to negative emotional terms was related to fewer externalizing, internalizing, and behavior problems in children. Maternal attribution of emotions to children was associated with lower negative social self in children. Maternal explanation of children’s emotions was linked to fewer externalizing problems and lower negative social self in children, and maternal reconfirmation of the explanations was related to fewer externalizing and behavioral problems in children. In contrast, maternal attribution of emotions to other people was associated with more externalizing problems and higher negative social self in children of both cultures. Some important cultural differences emerged. Chinese-American mothers’ mention of negative emotional terms was linked to lower negative social self in children, and Chinese-American mothers’ reconfirmation of explanation was related to more socially adaptive behaviors in children. No such relations were found in the European-American sample. The findings underscore the importance of family emotional reminiscing for children’s long-term well-being and the role of culture in shaping the process.

## Introduction

Parent–child reminiscing serves important adaptive functions, helping children build a sense of self, strengthen social relations, learn lessons from the past, and regulate emotions (Fivush et al., 2003; Wang, 2004; Wang and Fivush, 2005; Kulkofsky and Koh, 2009; Kulkofsky et al., 2009; Fivush, 2013). In particular, when children experienced emotionally negative events, the emotion regulation function of parent–child reminiscing is critical to children’s mental health. More common than not, although children may remember and recount what happened, parents often provide guidance for children to interpret and evaluate these emotionally negative experiences and thus regulate the aversive effects (Fivush et al., 2003). Such interpretative, evaluative, and regulatory efforts during emotional reminiscing are key to children’s mental health (e.g., Fivush et al., 2006; Pasupathi, 2013). Yet different cultures embrace different values toward emotions (Yang and Wang, 2019). Parents in different cultures may discuss emotionally negative events with their children in varied ways, which may hold different implications for children’s mental health. Moreover, most cross-cultural studies to date have focused on concurrent, rather than longitudinal, effects of parent–child emotional reminiscing on children’s mental health. Against this backdrop, the present study examined the long-term effects of mother–child reminiscing of emotionally negative events on children’s mental health in European-American and Chinese-American families.

### Mother–Child Reminiscing of Emotionally Negative Events

Mother–child reminiscing of emotionally negative events provides children with a meaning-making framework to interpret, evaluate, and regulate emotions. Specifically, these conversations involve not only discussions of what happened, but also focus on feeling states and causal explanations of the emotions that children experienced in the past event. Sales et al. (2003) observed that when mothers engaged in emotional reminiscing with their preschoolers aged 3–5, both mothers and children mentioned more negative emotions and causal explanations of the emotions when discussing a negative event about an injury-related emergency room visit than when discussing a positive event. Ackil et al. (2003) observed how mothers talked to their children aged 2.6 to 11.8 about a devastating tornado. They found that even at 4-month post-tornado, the conversations included more references to negative emotions and causes of emotions experienced, when compared with reminiscing about non-traumatic events. This pattern of mother–child emotional reminiscing persisted another 6months later (i.e., 10months after the tornado), which suggests an enduring focus on feeling states and causal explanations of the emotions experienced when mothers and children discuss traumatic events.

Not unlike highly stressful or traumatic events such as emergency room visits or devastating tornados, mother–child reminiscing of day-to-day stressors show similar characteristics of meaning-making. Burch et al. (2004) observed that 3-year-old children provided more interpretations of what happened, including references to their feelings and causal explanations for emotions experienced, when they were reminiscing with their mothers’ day-to-day negative events than nonnegative events. Likewise, Fivush et al. (2003) found that mothers attributed emotional states to the individuals involved when discussing daily events in which children experienced anger, and highlighted causal information when recollecting everyday events that elicited sadness, anger and fear, with their 4-year-old children.

Collectively, the findings suggest that regardless of the nature of emotionally negative events discussed (i.e., highly stressful, traumatic or mundane), mothers and children tend to focus on the emotions experienced and provide causal explanations for why those emotions were experienced. These characteristics of emotional reminiscing of negative events are pertinent to meaning-making whereby mothers scaffold children to interpret, evaluate, and regulate the negative emotions experienced. Nonetheless, the effect of such reminiscing and pertaining meaning-making on children’s long-term mental health has yet to be examined.

### Relations to Mental Health

There is a considerable range of mental health outcomes in relation to the meaning-making of emotionally negative experiences (Greenhoot and McLean, 2013). The outcomes include the reduction or absence of emotional and psychological symptoms, as well as enhancement or presence of normative attributes (e.g., prosocial behaviors) and desirable personality attributes. Studies have shown that mother–child reminiscing of emotionally negative events is predictive of various mental health outcomes in children.

One set of outcomes pertains to social well-being. For example, when comparing mother–child reminiscing of emotionally positive and negative events, Laible (2011) found that it was the reminiscing of negative events that were related concurrently to preschool children’s emotional and relational understanding. In particular, in-depth discussion of negative emotions predicted higher levels of emotional understanding in preschool children, and validation of negative emotions predicted higher levels of prosocial representations of relationships in preschool children.

Another set of outcomes is emotional well-being, including both internalizing and externalizing behaviors. Laible and Song (2006) compared discussions of negative and positive emotions during reminiscing and storybook-telling between mothers and their preschool children. She found that only discussion of negative emotions during reminiscing predicted concurrently lower levels of aggression in children. It was noted that discussion of negative emotions was summed across reminiscing of emotionally negative and positive events in this study. Although the effects of reminiscing about positive and negative events could not be teased apart, the finding underscores that discussion of negative emotions may contribute to lower levels of behavioral problem in children.

Notably, the nature of the emotionally negative events may influence how reminiscing affect children’s emotional well-being. Sales and Fivush (2005) asked mothers to discuss with their children aged 8–12 one chronic experience and one acute stressful experience related to the child’s asthma. Chronic experiences included events concerning the day-to-day management of the illness, and acute stressful experiences included events such as an unexpected asthma attack that required emergency room treatment. Children’s concurrent emotional well-being was measured. Mothers provided more causal explanations for the chronic event than the acute event. Importantly, for discussions of the chronic event, mothers who mentioned more emotions had children who showed fewer internalizing and externalizing behaviors, and mothers who provided more causal explanations had children who showed fewer externalizing behaviors. Conversely, for discussions of the acute event, there was no relation between maternal discussion and explanation of emotions and children’s mental health. It was further observed that children who mentioned more emotions, but without explanations of those emotions, showed more internalizing behaviors.

Taken together, the findings suggest that discussion of emotions and provision of causal explanations during reminiscing of emotionally negative events, especially those that are experienced in the day-to-day context, are associated with children’s concurrent positive mental health. However, in face of highly stressful events, the search for meaning may be difficult (Fivush et al., 2008). Furthermore, when feeling states are mentioned without causal explanations, children seem worse off in their emotional well-being. Extant studies have thus provided some evidence for the effects of mother–child reminiscing of emotionally negative events on children’s mental health. Nonetheless, these studies have focused on concurrent effects and have largely been conducted with European-American families and have not taken into consideration the influence of the larger cultural context (Wang, 2013a; Yang and Wang, 2019).

### Mother–Child Reminiscing in the Cultural Context

Different cultures hold different values towards emotion. In European-American culture where individuality is valued, talking about emotion is viewed as a direct expression of the self and an affirmation of the significance of the individual (Wang, 2013a). European-American mothers believe that it is important to help children convey and articulate their emotions so that they could get their needs met (Chao, 1995). Conversely, in Chinese culture where relationships are valued, emotion (especially negative emotion) is regarded as disruptive to social harmony and expressions of emotions are often discouraged (Chao, 1995; Wang, 2013a). Instead, sensitivity to others’ emotions is encouraged (Wang, 2001; Yang and Wang, 2019). Furthermore, as emotions are often viewed as a consequence of children’s social acts, Chinese parents tend to focus on instilling moral rules, discipline, and proper behaviors in children during emotional reminiscing (Wang, 2001; Fivush and Wang, 2005; Wang and Fivush, 2005). These Chinese values are frequently upheld by first-generation Chinese immigrant parents such as those in the USA, who often strive to maintain their heritage culture and socialize their children accordingly (Chang and Karl Kwan, 2009; Kim, 2009). For example, Koh et al. (2009) observed that both Chinese-American fathers and mothers showed higher levels of ethnic values than mainstream American values. Wang (2007) also observed that Chinese-American mothers upheld Chinese interdependent values as much as Chinese mothers in Beijing did.

Mother–child reminiscing of emotional experiences, particularly negative emotional experiences, has largely mirrored the respective cultural values towards emotions (Wang, 2001; Fivush and Wang, 2005; Wang and Fivush, 2005; Wang et al., 2010). Both European-American and Chinese mothers and children tend to use more negative emotion words than positive emotion words, but Chinese mothers and children use more negative emotion words than European-American mothers and children, as negative emotions are deemed to serve a didactic function. Although mothers of the two cultures similarly attribute emotions to children during the reminiscing, European-American children make more attributions of emotions to themselves than Chinese children, whereas Chinese children make more attribution of emotions to other people than European-American children. Furthermore, European-American mothers and children make more causal explanations for the emotions experienced by children and other people than Chinese mothers and children. In contrast, Chinese mothers and children engage in more didactic talk that focuses on moral rules, discipline, and behavioral expectations, than European-American mothers and children. Notably, in line with their emphasis on preserving heritage culture and ethnic identity in childrearing (Chang and Karl Kwan, 2009; Kim, 2009; Koh et al., 2009), first-generation Chinese-American mothers reminisce negative emotional events with their children more in line with traditional Chinese values than do Chinese mothers in Beijing (Wang, 2013b).

In a similar vein, although meaning-making of emotionally negative events during mother–child reminiscing may generally facilitate positive well-being in children, culture may play a role in the relation. The person-culture-fit framework has underscored the congruence between individual characteristics and cultural norms in predicting psychosocial adjustment (Lerner, 2002; Caldwell-Harris and Ayçiçegi, 2006; Chen, 2018). For example, Wang and colleagues (Wang et al., 2018) found that detailed recall of autobiographical experiences, in congruence with American cultural values of individuality, was related to positive well-being in European-American adults and children. In contrast, detailed recall of one’s own experiences, incongruent with Chinese values of modesty and relationship orientation, was related to ill-being in Chinese adults and children. Similarly, Reese and colleagues found that recalling events of personal growth was related to well-being in European New Zealand adolescents, but not in Maori and Chinese New Zealand adolescents (Reese et al., 2017).

In sum, studies have revealed key characteristics of mother–child reminiscing of negative emotional experiences in the European-American and Chinese cultural contexts, which are in line with European-American and Chinese cultural values, respectively (Wang, 2013a; Yang and Wang, 2019). An important question remains with regard to the effects, particularly the long-term effects, of mother–child emotional reminiscing on children’s mental health across cultures. The person-culture-fit framework and related empirical findings (Reese et al., 2017; Wang et al., 2018) suggest that culture may moderate the relations between mother–child reminiscing of emotionally negative events and children’s mental health. In particular, culture-specific ways of mother–child reminiscing, reflective of cultural values, may serve as key meaning-making channels and relate to positive mental health in children.

### The Present Study

The present study examined the long-term relations of early mother–child reminiscing of emotionally negative events to children’s later mental health in the European-American and Chinese-American cultural contexts. To assess how mothers generally engage in emotional reminiscing with their children about negative events, we asked mother–child dyads to discuss one day-to-day event in which the child experienced negative feelings, when children were 4.5years of age. This method is based on prior research that shows maternal reminiscing style as a stable individual characteristic across contexts (Haden, 1998; Wang et al., 2000; Wang, 2001; Fivush et al., 2006). To assess the long-term associations of mother–child emotional reminiscing with children’s well-being, when children were age 7, children’s mental health was measured, including social well-being as assessed in terms of socially adaptive behaviors (e.g., social skills), and emotional well-being as assessed in terms of internalizing problems (e.g., depressive mood), externalizing problems (e.g., conduct problems), and behavioral problems (e.g., withdrawal).

In line with findings from prior studies (Wang, 2001; Fivush and Wang, 2005; Wang and Fivush, 2005; Wang et al., 2010), we expected that European-American mothers and children would provide more causal explanations about children’s and other people’s emotions and would make more attributions of emotions to children than Chinese-American mother–child dyads. Conversely, Chinese-American mothers and children would mention more negative emotions, engage in more didactic talk, and make more attributions of emotions to other people than European-American mother–child dyads. Furthermore, earlier studies comparing European-American and Chinese children in the preschool and middle childhood years found that European-American children scored higher on externalizing problems, while Chinese children scored higher on internalizing problems, although overall, there were considerable similarities in the problems exhibited by children in the two cultures (Weine et al., 1995; Liu et al., 2011). Accordingly, we expected to observe similar patterns of findings in the mental health of European-American and Chinese-American children in our study.

Pertaining to the relations between mother–child emotional reminiscing and children’s mental health, we expected that, in line with the general literature as well as prior cross-cultural research (e.g., Lerner, 2002; Caldwell-Harris and Ayçiçegi, 2006; Fivush et al., 2008; Reese et al., 2017; Chen, 2018; Wang et al., 2018), meaning-making of negative emotional experiences would facilitate mental health in children of both cultures and in the meantime show culture-specific patterns. Specifically, we predicted that (1) references to negative emotions would be related to positive mental health indexes in both groups of children, but the relations might be stronger in Chinese-American children than European-American children given the didactive value placed on negative emotions in Chinese culture (Fivush and Wang, 2005); (2) attributions of emotions to children and explanations about the causes of children’s emotions would be associated with positive mental health indexes in children of both groups, but the relations may be greater in European-American children than Chinese-American children given the great emphasis on facilitating emotional understanding in American childrearing (Wang, 2001; Fivush and Wang, 2005; Wang and Fivush, 2005; Wang et al., 2010); (3) attributions of emotions to other people and explanations about the causes of other people’s emotions would be linked to positive mental health indexes in Chinese-American children, given the Chinese cultural emphasis on interdependence and sensitivity to others’ feedings (Wang, 2001); and (4) didactic talk would be related to positive mental health indexes in Chinese-American children, given the emphasis on moral rectitude and behavioral discipline in Chinese values and socialization practices (Wang, 2001; Wang and Fivush, 2005). Lastly, although there has been no suggestion in the mother–child reminiscing literature, it has been shown that in children’s independent recount of a traumatic experience, references to positive emotions are related to better emotional well-being across time (Sales et al., 2005) and that redemption in the reconstruction of negative experiences is predictive of well-being (McAdams and Guo, 2015). We thus predicted that mother–child references to positive emotions during reminiscing of a negative event would be related to positive mental health indexes in children of both cultures.

## Materials and Methods

### Participants

This study was part of a larger longitudinal project on autobiographical memory development which included data collection across five time-points, with children’s ages spanning from 3 to 7.25 years. This study utilized the data at Time 3, when children were 4.5years old and Time 4, when children were 7years old. Sample attrition between these two time points was 48%, due to family relocation and loss of contact.

Specifically, the final sample of participants in this study comprised of 33 European-American (19 boys) and 22 Chinese-American (10 boys) children and their mothers. All families were recruited from a university town and suburban areas in upstate New York. Recruitment was conducted through local preschools and words of mouth. All families were middle-class, with the majority of the mothers and fathers having a college education and beyond. The Chinese-American families were originally from Mainland China, Hong Kong, and Taiwan. All the Chinese-American mothers were first-generation immigrants in the United States. All the Chinese-American children were born in the USA, except for two, who came to the USA at around age 2. Mothers provided informed consent for their children’s participation.

### Procedure and Measures

The general data collection procedure was the same across time points. Two female researchers visited mothers and children in their homes. The main researcher worked through the study procedure with the mothers and children and the second researcher took charge of the audio and video recordings. The main researcher was always a native of the culture of the family visited and spoke the native language. That is, a European-American researcher served as the main researcher during the home visits to European-American families and an English-Chinese bilingual Chinese researcher served as the main researcher during the home visits to Chinese-American families. Specific to the Chinese-American families, mothers and children were asked to speak in the language they normally spoke at home, which could be English, Chinese, or a mixture of both. All materials were prepared in both English and Chinese. A translation and back-translation procedure was conducted to ensure language equivalence in literal and sense meanings. Generally, each visit comprised of a mother–child segment where the mother–child pair was asked to engage in tasks including memory-sharing, a researcher-child segment where the main researcher worked through the various tasks with the child, and a mother segment where the mother responded to a battery of questionnaires. The mother–child and researcher-child segments were audio- and video-recorded. Each visit lasted for about 1.5–2h. At the end of each visit, the child was given a small gift, and the mother was given a $30 gift card in appreciation of their participation. The measures relevant to this study are described below.

#### Language

When children were aged 4.5, mothers responded to the shorten version of the Child Development Inventory (Ireton, 1992). This measure assesses children’s language production and comprehension. Mothers responded to 100 items with the total possible score ranging from 0 to 100. A higher score indicates a higher level of language production and comprehension. In this sample, Cronbach’s *α* was 0.86.

#### Emotion Talk

When children were aged 4.5, mothers were asked to talk to children about a specific, one-time event that mother and child experienced together. To ensure discussion of negative emotions, mothers were asked to nominate an event that was emotionally negative to the child, following prior research (Fivush et al., 2003; Wang and Fivush, 2005; Wang et al., 2010). Mothers were asked to choose an event that took place within the last 2months so that children still had memories of it. Mothers were asked to discuss the event with their children as they normally would and for as long as they wanted. During the conversation, the researchers stayed in the room to work on the audio- and video-recording unobtrusively. Each conversation lasted for approximately 10min.

#### Behavior Assessment System for Children-Second Edition (BASC-2)

When children were aged 7, mothers responded to the Parent Rating Scales of BASC-2 (Reynolds and Kamphaus, 2005). This is a 160-item multidimensional measure that assesses four dimensions of problems and behaviors in children, including internalizing problems (comprise of anxiety, depression, and somatization, e.g., “Is negative about things”), externalizing problems (comprise of hyperactivity, aggression, and conduct problems, e.g., “Hits other children”), behavioral problems (comprise of atypicality, withdrawal, and attention problems, e.g., “Acts confused”), and socially adaptive behaviors (comprise of adaptability, social skills, leadership, activities of daily living, and functional communication, e.g., “Tries to bring out the best in other people”), in addition to “critical items” that hold clinical significance. Only the items that assess the four dimensions of problems and behaviors in children were used in the present study. The number of items in the four subscales ranges from 6 to 14. Mothers responded to the items on a four-point Likert scale (1=Never to 4=Always). A higher score in each subscale indicates higher level of the problems or behaviors. In this sample, Cronbach’s *α*s for the four subscales ranged from 0.74 to 87.

#### Negative Social Self

As this study included Chinese-American children, an Asian-salient dimension of depression – negative social self was included to provide a comprehensive coverage of internalizing problems relevant to the study’s participants. When children were aged 7, mothers of both groups responded to the Asian Children Depression Scale-Caretaker Version. This 20-item measure was adapted from the original child version (Koh et al., 2007). It comprises of three universally recognized dimensions of depression: negative affect and cognitive dysfunction (e.g., “I feel sad”), loss of interest (e.g., “I feel that nothing is fun”), and psychosomatic manifestations (e.g., “My body feels painful”), as well as a culturally salient dimension of depression: negative social self (e.g., “I feel that I can no longer make my parents happy”). Mothers responded to the items on a five-point Likert scale (1=Not at all like my child to 5=Most like my child). Only the negative social self subscale was utilized in the current study. This subscale originally comprised of six items, but one item was dropped due to a negative item-total correlation with the whole scale. A higher score on this subscale indicates a higher level of negative social self. In this sample, Cronbach’s *α* was 0.46. Note that Cronbach’s *α* is highly sensitive to test length, whereby the value is reduced with short test length (e.g., Streiner, 2003). For example, Weisz and colleagues utilized the Contingency, Competence and Control Probes, which was a measure with three subscales and each of the subscales had four items, and reported Cronbach’s *αs* for the three subscales in the range of 0.39–0.65 (Weisz et al., 1987, 1989). Given that the negative social self subscale had five items and the Cronbach’s *α* was 0.46, it is within the range reported in the literature.

### Coding

Mother–child conversations were coded using Noldus’s program The Observe*R*^®^ 5.0. This is a coding program whereby coders view the video materials and score the codes directly on the computer (Noldus, 2003). Coding was conducted in the original languages. Proposition, defined as a subject-verb construction (Fivush et al., 1995), was the coding unit, unless otherwise noted. Each unique or implied verb in an independent clause forms a new propositional unit. For example, “You were sad” was one proposition and “You were sad and crying” was two. All variables were coded for frequency. This allowed us to examine not only the presence of the codes but also how frequent and thus how important they were in the meaning-making process. Mothers’ and children’s utterances were coded separately and into one of the following mutually exclusive and exhaustive categories.

*1. Emotion terms:* Mothers’ and children’s utterances of specific emotion terms, including emotional states (e.g., *happy*) and emotional behaviors (e.g., *crying*). Positive and negative emotion terms were coded separately. The coding unit for this variable was emotion term.
*2a. Attribution-child:* Mothers’ utterances that ascribed emotional states or reactions to their children (e.g., M: *You were scared, weren’t you?*); children’s utterances that ascribed emotional states or reactions to themselves (e.g., C: *I was mad*.)
*2b. Mothers’ reconfirmation of attribution:* In a three-utterance sequence, mothers’ utterances that reconfirmed the emotional states or reactions ascribed to their children (e.g., M: You were scared. C: Yeah. Mother reconfirmed: *Yeah, you were scared*.) The coding unit for this variable was instance of occurrence of reconfirmation.
*2c. Attribution-others*: Mothers’ and children’s utterances about other people’s emotional states or reactions (e.g., M: *Grandma was angry, wasn’t she?*/C: *Daddy was mad*.)
*3a. Explanation-child:* Mothers’ utterances about the causes of their children’s emotional states or reactions (e.g., M: *You were scared because daddy was fierce*; M: *Why were you sad?*); children’s utterances about the causes of their own emotions (e.g., *I cried because I did not like it*.)
*3b. Mothers’ reconfirmation of explanation*: In a three-utterance sequence, mothers’ utterances that reconfirmed the causes of their children’s emotional states or reactions (e.g., M: Why were you sad? C: I lost my dolly. Mother reconfirmed*: Yeah, you did*.) The coding unit for this variable was instance of occurrence of reconfirmation.
*3c. Explanation-others*: Mothers’ and children’s utterances about the causes of other people’s emotional states or reactions (e.g., M: *Daddy was mad because you were naughty*./C: *I did not listen to grandma, so she was angry*.)
*4. Didactic content*: Mothers’ and children’s utterances about moral standards, social norms, or behavioral expectations and disciplines (e.g., M: *It was wrong of you to make grandma angry*./C: *Children should listen to their parents*.)

English-speaking and English-Chinese bilingual research assistants coded the datasets in English and Chinese, respectively. All coders were unaware of the study hypotheses. Repeated joint coding sessions were held to ensure that the same definitions were followed by all the coders. Intercoder reliability was assessed for 20% of the data from each subsample. Kappas ranged from 0.76 to 0.93 for the European-American sample and 0.78–0.89 for the Chinese-American sample.

## Results

Preliminary analyses revealed no systematic effects involving age, gender, and language; these variables were therefore not considered further. For the mental health measures, missing data were replaced with series means.

### Types of Negative Events Discussed

For European-American mother–child pairs, commonly discussed negative events were injuries and medical procedures (30.3%), conflicts with or scolding from parents (18.2%), disappointments (15.2%), and conflicts with siblings or peers (12.1%). Other negative events discussed were about death (9.1%), separation from caregivers and others (9.1%), scary things (3.0%), and losing a special object (3.0%). For Chinese-American mother–child pairs, commonly discussed negative events were conflicts with siblings or peers (31.8%), conflicts with or scolding from parents (22.7%), and injuries and medical procedures (22.7%). Other negative events discussed included disappointment (9.1%), new environment (9.1%), and scary things (4.5%). Thus, mother–child pairs of both cultural groups talked about a wide range of events. Particularly notable, Chinese-American mother–child pairs (54.5% total) were more likely than European-American mother–child pairs (30.3% total) to discuss conflicts with parents, siblings or peers, *χ*^2^(1, *N*=55)=3.15, *p*=0.08, *φ*=0.24.

### Mother–Child Emotional Reminiscing

The means for the emotion talk categories were generally low (<1). Mothers’ and children’s utterances were thus dummy coded and analyzed as categorical data. For each coded variable, mothers and children received 1 if they made any utterance and received 0 if they did not make any utterance in relation to that variable. Due to low proportions of children who made utterances about other’s emotions (i.e., attribution-others) and the causes of others’ emotions (i.e., explanation-others), these variables were not analyzed further. Table 1 shows the proportions of mothers and children who made utterances for the different emotion talk variables by cultural group. To examine cultural differences in the emotion talk variables, binary logistic regression analyses using Generalized Linear Model were conducted, with culture as the predictor for each of the emotion talk variables (e.g., mothers’ negative emotion terms).

**Table 1:** Percentages of mothers and children who provided responses for the emotion talk variables by culture.

Emotional reminiscing	Mothers			Children		
	EA	CI	Total	EA	CI	Total
Emotion terms: Negative	52	77	62	27	23	26
Emotion terms: Positive	33	23	29	12	14	13
Attribution-child	52	64	56	52	27	42
Mothers’ reconfirmation of attribution	30	18	26	–	–	–
Attribution-others	27	9	20	15	5	11
Explanation-child	67	64	66	67	50	60
Mothers’ reconfirmation of explanation	30	18	26	–	–	–
Explanation-others	15	9	13	6	0	4
Didactic talk	9	46	24	9	32	18

*EA=European-American; CI=Chinese-American*.

#### Mothers’ Utterances

Chinese-American mothers were more likely than European-American mothers to utter negative emotion terms, Wald *χ*^2^(1, *N*=55)=3.56, *p*=0.06, *φ*=0.25, and didactic content, Wald *χ*^2^(1, *N*=55)=8.17, *p*<0.01, *φ*=0.39. No other effects reached significant difference.

#### Children’s Utterances

European-American children were more likely than Chinese-American children to ascribe emotional states or reactions to themselves, Wald *χ*^2^(1, *N*=55)=3.10, *p*=0.08, *φ*=0.24. Chinese-American children were more likely than European-American children to utter didactic content, Wald *χ*^2^(1, *N*=55)=4.12, *p*<0.05, *φ*=0.27. No other effects reached significant difference.

### Children’s Mental Health

To examine cultural differences in children’s mental health, independent sample t-tests were conducted. Table 2 shows the means and standard deviations of the variables examined. Based on mothers’ reports, European-American children showed more externalizing problems than Chinese-American children, *t*(53)=2.66, *p*<0.05, *d*=0.75. No other effects reached significance.

**Table 2:** Means and standard deviations of mental health variables by culture.

Mental health	European-American	Chinese-American
	*M* (*SD*)	*M* (*SD*)
Internalizing problems^a^	1.52 (0.19)	1.54 (0.27)
Negative social self^b^	1.07 (0.14)	1.08 (0.16)
Externalizing problems^a^	1.72 (0.30)	1.52 (0.23)
Behavioral problems^a^	1.62 (0.25)	1.66 (0.32)
Adaptive behaviors^a^	3.03 (0.35)	2.88 (0.40)

a *Four-point Likert scale (1=Never to 4=Always)*.

b *Five-point Likert scale (1=Not at all like my child to 5=Most like my child)*.

### Mother–Child Emotional Reminiscing and Children’s Mental Health

To examine the longitudinal relations of mother–child emotional reminiscing to children’s mental health and whether the relations were moderated by culture, univariate ANOVAs were conducted, with culture, emotion talk (i.e., each emotion talk variable), and culture x emotion talk interaction predicting each mental health dimension (e.g., externalizing problems). Significant interaction effects were followed up with focused comparisons.

#### Mothers’ Utterances

##### Emotion terms: Negative

There was a marginally significant main effect of maternal utterances of negative emotion terms on child internalizing problems, *F*(1, 51)=3.18, *p*=0.08, *η_p_*^2^=0.06, *M(SD)*_Talk_=1.50 (0.20), *M*(*SD*)_Not Talk_=1.58 (0.26). There were main effects of mothers’ utterances of negative emotion terms on child externalizing problems, *F*(1, 51)=5.14, *p*<0.05, η*_p_*^2^=0.09, *M(SD)*_Talk_=1.56 (0.27), *M*(*SD*)_Not Talk_=1.78 (0.26), and child behavioral problems, *F*(1,51)=5.73, *p*<0.05, *η_p_*^2^=0.10, *M(SD)*_Talk_=1.57 (0.25), *M*(*SD*)_Not Talk_=1.73 (0.30). Mothers who made references to negative emotions had children with fewer internalizing problems, externalizing problems, and behavioral problems than mothers who did not.

**Figure 1:**
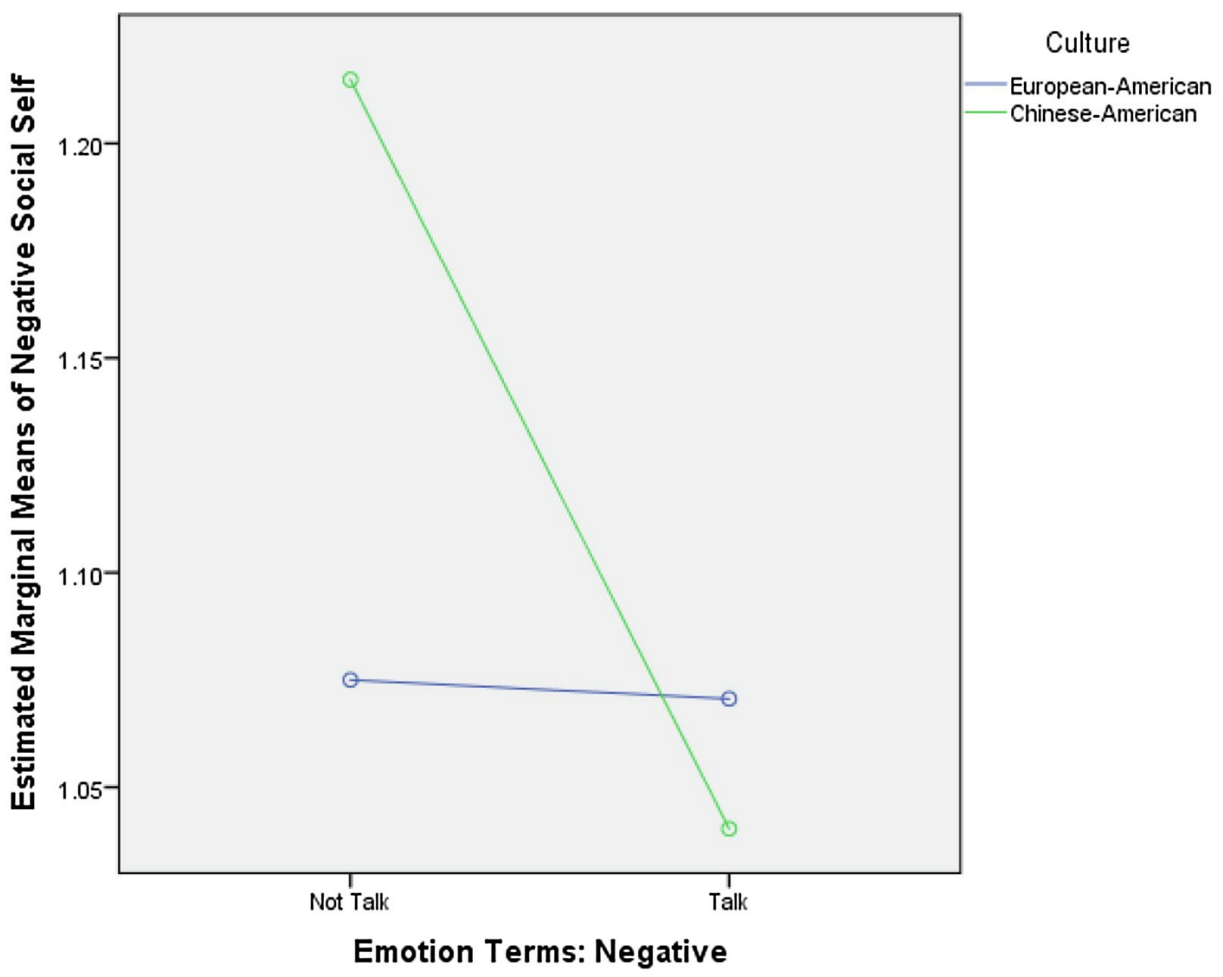
Culture by mothers’ negative emotion terms interaction effects on children’s negative social self. ^*^p<0.05.

Furthermore, there was a main effect of maternal utterances of negative emotion terms on child negative social self, *F*(1, 51)=4.22, *p* <0.05, *η_p_*^2^ =0.08, qualified by a marginally significant Culture x Negative emotion terms interaction, *F*(1, 51)=3.81, *p* =0.06, *η_p_*^2^ =0.07. Chinese-American mothers who talked about negative emotions had children with lower negative social self than mothers who did not, *F*(1, 20)=5.99, *p* <0.05, *η_p_*^2^ =0.23, *M(SD)*_Talk_ =1.04 (0.08), *M*(*SD*)_Not Talk_ =1.21 (0.27). This effect was not found in the European-American sample. Figure 1 presents the interaction effect.

##### Attribution-Child

There was a marginally significant main effect of maternal attribution of emotional states or reactions to their children on child negative social self, *F*(1, 51)=3.53, *p*=0.07, *η_p_*^2^=0.07, *M(SD)*_Talk_=1.05 (0.10), *M*(*SD*)_Not Talk_=1.11 (0.18). Mothers who ascribed emotional states or reactions to their children had children with lower negative social self than mothers who did not.

##### Attribution-Others

There was a marginally significant main effect of maternal attribution of emotional states or reactions to other people on child externalizing behaviors, *F*(1, 51)=2.71, *p*=0.106, *η_p_*^2^=0.05, *M(SD)*_Talk_=1.80 (0.31), *M*(*SD*)_Not Talk_=1.60 (0.27). There was also a main effect of maternal attribution of emotional states or reactions to other people on child negative social self, *F*(1, 51)=6.24, *p*<0.05, *η_p_*^2^=0.11, *M(SD)*_Talk_=1.15 (0.18), *M*(*SD*)_Not Talk_=1.06 (0.13). Interestingly, mothers who ascribed emotional states or reactions to other people had children with more externalizing problems and higher negative social self than mothers who did not.

##### Explanation-Child

There was a marginally significant main effect of maternal explanations of the causes of children’s emotional states or reactions on child externalizing behaviors, *F*(1, 51)=3.49, *p*=0.07, *η_p_*^2^=0.06, *M(SD)*_Talk_=1.59 (0.26), *M*(*SD*)_Not Talk_=1.74 (0.32). There was also a main effect of maternal explanations of the causes of children’s emotional states or reactions on child negative social self, *F*(1, 51)=9.74, *p*<0.01, *η_p_*^2^=0.16, *M(SD)*_Talk_=1.04 (0.09), *M*(*SD*)_Not Talk_=1.15 (0.20). Mothers who explained the causes of their children’s emotional states or reactions had children with fewer externalizing problems and lower negative social self than mothers who did not.

##### Mothers’ Reconfirmation of Explanation

There were main effects of maternal utterances that reconfirmed the causes of their children’s emotional states or reactions on child externalizing problems, *F*(1,51)=4.62, *p*<0.05, *η_p_*^2^=0.08, *M(SD)*_Talk_=1.51 (0.20), *M*(*SD*)_Not Talk_=1.69 (0.30), and child behavioral problems, *F*(1, 51)=5.62, *p*<0.05, *η_p_*^2^=0.10, *M(SD)*_Talk_=1.48 (0.28), *M*(*SD*)_Not Talk_=1.69 (0.26). Mothers who reconfirmed the causes of their children’s emotional states or reactions had children with fewer externalizing problems and behavioral problems than mothers who did not.

**Figure 2:**
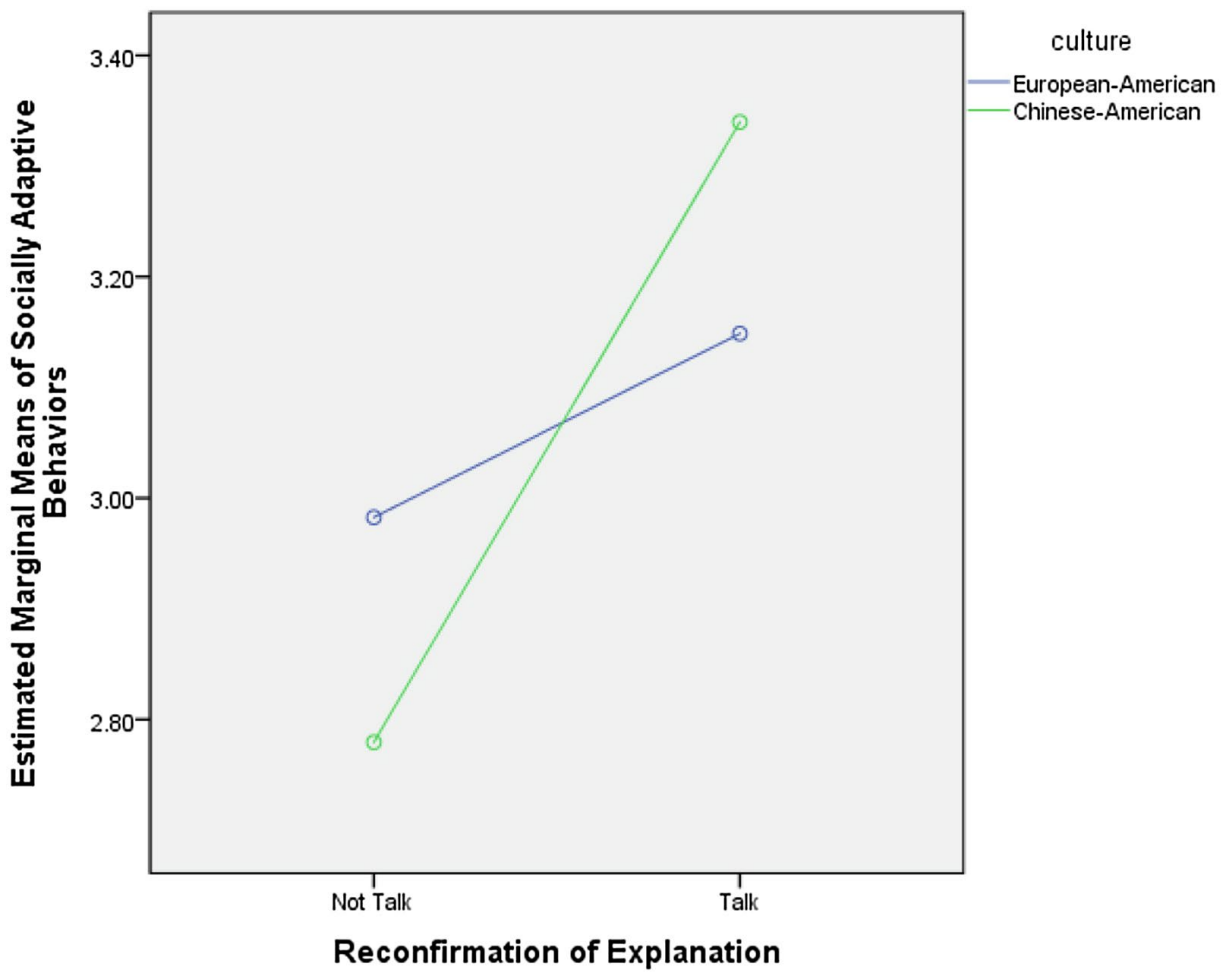
Culture by mothers’ reconfirmation of explanation interaction effects on children’s socially adaptive behaviors. ^**^p<0.01.

Furthermore, there was a main effect of maternal utterances that reconfirmed the causes of their children’s emotional states or reactions on child socially adaptive behaviors, *F*(1, 51)=10.00, *p* <0.01, η*_p_*^2^ =0.16, qualified by a marginally significant Culture x Reconfirmation of explanation interaction, *F*(1, 51)=2.95, *p* =0.09, *η_p_*^2^ =0.06. Chinese-American mothers who reconfirmed the causes of their children’s emotional states or reactions had children with more socially adaptive behaviors than mothers who did not, *F*(1, 20)=8.94, *p* <0.01, *η_p_*^2^ =0.31, *M(SD)*_Talk_ =3.34 (0.60), *M*(*SD*)_Not Talk_ =2.78 (0.27). No such effect was found in the European-American sample. Figure 2 presents the interaction effect.

##### Other Utterances

There were no effects of maternal utterances of positive emotion terms, reconfirmation of the emotional states or reactions ascribed to children, utterances about causes of other people’s emotional states or reactions, and utterances about moral standards, social norms, or behavioral expectations and disciplines (i.e., didactic content) on children’s mental health.

#### Children’s Utterances

##### Explanation-Child

There was a marginally significant main effect of children’s utterances about the causes of their emotional states or reactions on negative social self, *F*(1, 51)=2.91, *p*=0.09, *η_p_*^2^=0.05, *M(SD)*_Talk_=1.05 (0.12), *M*(*SD*)_Not Talk_=1.12 (0.17). Children who uttered about the causes of their emotional states or reactions had lower negative social self than children who did not.

##### Other Utterances

All other children’s utterances, including utterances of negative and positive emotion terms, utterances that ascribed emotional states or reactions to themselves and other people, utterances about causes of other people’s emotional states or reactions as well as utterances about moral standards, social norms, or behavioral expectations and disciplines (i.e., didactic content) did not have effects on their mental health.

## Discussion

Albeit a growing literature that examines the relations between mother–child reminiscing of emotionally negative events and children’s mental health, studies that investigate the cultural and longitudinal effects have been scanty. The present study is the first to examine the long-term relations of mother–child reminiscing of emotionally negative events to children’s mental health in European-American and Chinese-American families. In line with our expectations, family meaning-making largely predicted positive mental health in children, although culture also plays a role. The findings provide important insights regarding the long-term effects of mother–child emotional reminiscing on children’s psychosocial adjustment in the cultural context.

Mothers and children of the two cultures discussed similar negative events during reminiscing. Most of the negative events surrounded conflicts with social others, including parents, siblings, and peers, as well as medical and disappointing experiences. This reflects fairly common negative life events experienced by children of this age. Interestingly, Chinese-American mother–child pairs were more likely than European-American mother–child pairs to discuss social conflicts, consistent with prior cross-cultural findings (Fivush and Wang, 2005). Operating in a cultural milieu that emphasizes social connectedness and relational harmony (Chao, 1995; Wang, 2013a), social interactions, particularly negative ones, may warrant more concern and discussion and are therefore more commonly discussed during mother–child reminiscing in Chinese than in European-American families.

When reminiscing about emotionally negative events, Chinese-American mothers were more likely than European-American mothers to mention negative emotions and to engage children in didactic talk. Chinese-American children were also more likely than European-American children to engage in didactic talk. These findings are consistent with our predictions. They confirm those in earlier studies (Wang, 2001; Fivush and Wang, 2005; Wang and Fivush, 2005) and suggest that mother–child reminiscing of emotionally negative events serves a didactic function in the Chinese context. Given their cultural emphasis on moral rectitude and social harmony, Chinese mothers are particularly concerned with helping their children learn appropriate emotional responses and regulations and instilling in children proper behaviors and self-control (Wang and Fivush, 2005; Wang, 2013a). Correspondingly, Chinese children appear to exhibit similar concerns as their mothers do about responding and behaving appropriately when discussing emotionally negative events. Also as expected and in line with prior research (Fivush and Wang, 2005; Wang et al., 2010), European-American children were more likely than Chinese-American children to attribute emotions to themselves. This aligns with the European-American cultural value that emphasizes individuality, where discussion of one’s own emotions is a way to affirm the authenticity of the self (Chao, 1995; Wang, 2013a).

European-American and Chinese-American children showed similar mental health indexes across the multiple measures, as expected. Both groups of children displayed similar levels of internalizing problems, negative social self, behavioral problems, and socially adaptive behaviors. There was only one exception, whereby European-American children exhibited more externalizing problems than did Chinese-American children. This pattern of findings largely conforms to earlier observations (Weine et al., 1995; Liu et al., 2011).

With regards to the relations between mother–child emotional reminiscing and children’s long-term mental health, we found that, independent of culture, mothers who mentioned negative emotions had children with fewer internalizing problems, externalizing problems, and behavioral problems than mothers who did not, as predicted. Fivush et al. (2003) argued that discussions of negative feeling states help children learn appropriate ways of responding to the emotionally negative events and regulating their emotions. This reminiscing characteristic is therefore associated with better mental health in children of both cultures. Furthermore, the link between maternal references to negative emotions and child mental health appeared stronger among Chinese-Americans than European-Americans, as expected. Chinese-American mothers who referenced negative emotions had children with lower negative social self when compared to mothers who did not. Discussions of negative feeling states serve a didactic function in the Chinese cultural context, where discipline and proper behaviors are emphasized as ways of regulating emotions (Wang, 2001; Fivush and Wang, 2005; Wang and Fivush, 2005). Accordingly, this reminiscing characteristic appears to have an additional positive effect on mental health in Chinese-American children.

Furthermore, mothers who ascribed emotional states or reactions to children had children with lower negative social self than mothers who did not, and that was the case for both cultures, as predicted. Mothers who ascribed the emotional states or reactions experienced in the negative events to children help them understand and regulate the emotions (Ackil et al., 2003; Fivush et al., 2003; Sales et al., 2003; Laible and Song, 2006). Through scaffolding children to understand and regulate their emotions, this reminiscing characteristic may promote better mental health in children. Similarly, as expected, mothers who explained the causes of the children’s emotional states or reactions had children with fewer externalizing problems and lower negative social self than mothers who did not. And mothers who reconfirmed the causes of the children’s emotional states or reactions had children with fewer externalizing problems and behavioral problems than mothers who did not. These findings are consistent with suggestions in the literature (Ackil et al., 2003; Fivush et al., 2003; Sales et al., 2003; Sales and Fivush, 2005; Laible and Song, 2006; Laible, 2011) that explaining the causes of children’s emotions and reconfirming those explanations are important meaning-making mechanisms for promoting better mental health in children.

As noted above, both European-American and Chinese-American mothers who reconfirmed the causes of the children’s emotional states or reactions had children with fewer externalizing problems and behavioral problems than mothers who did not. Yet, although contrary to our prediction, there was an additional relation in terms of having children with more socially adaptive behaviors when Chinese-American mothers reconfirmed the causes of their children’s emotional states or reactions compared to mothers who did not. Earlier studies have reported that European-American mothers tend to make more causal explanations for the emotions experienced by children than Chinese mothers (Wang, 2001; Wang and Fivush, 2005; Wang et al., 2010). However, contrary to our expectation, we did not find in the present study cultural differences in mothers’ causal explanations of their children’s emotions or mothers’ reconfirmation of those explanations. These findings may reflect acculturation in the Chinese-American mothers (Liem et al., 2000). When mothers offer causal explanations as well as reconfirmation of those explanations, the reminiscing serves critical meaning-making functions (Ackil et al., 2003; Fivush et al., 2003; Sales et al., 2003; Sales and Fivush, 2005; Wang and Fivush, 2005). These reminiscing patterns, therefore, exhibit positive relations to children’s mental health regardless of culture. Importantly, when Chinese mothers reconfirm those explanations, there appears to be an additional positive relation to children’s mental health.

Contrary to our prediction, mothers who ascribed emotional states or reactions to other people had children with more externalizing problems and higher negative social self than mothers who did not, regardless of culture. It appears that during reminiscing of children’s past negative experiences, highlighting to children others’ emotions, especially doing so without providing explanations, may not be conducive to the well-being of children. In other words, discussing *whose* emotions matters during emotional reminiscing of negative experiences: It is the discussion of the child’s emotions, not others’ emotions, that promote better mental health in children. This is so even in the Chinese context where sensitivity to other people’s feelings is encouraged (Wang, 2001, 2013b). It appears that children experienced in the past events negative emotions that need to be resolved, and reminiscing that helps them understand and regulate these emotions can promote positive mental health. Conversely, discussing other people’s feeling states in the past events, especially when not accompanied by explanations, might introduce social stress in children, which, in turn, can be associated with negative mental health. Notably, there were limited effects of children’s own emotional utterances during mother–child reminiscing when they were at age 4.5 on their own mental health when they were at age 7. This may be due to the young age when children were engaged in emotional reminiscing with their mothers, which does not appear to support the sustainable, long-term effects on their mental health.

The present study yielded important insights into the long-term effects of mother–child reminiscing of emotionally negative events on children’s mental health. Nonetheless, there are limitations to consider for future research. First, meaning-making of emotionally negative events comprises many dimensions, such as emotion talk, narrative coherence, and understanding of internal states (i.e., cognitions, emotions, and subjective perspectives; e.g., Fivush et al., 2008; Wang et al., 2010; Greenhoot and McLean, 2013; Pasupathi, 2013). Although it is important by and in itself, the present study focused on examining emotion talk and its various aspects. Furthermore, research has recognized that the aspects of mental health studied to date have been rather limited (Pasupathi, 2013). For example, internalizing and externalizing behaviors are commonly examined. The present study examined similar aspects of mental health. Future studies may extend the analyses to include narrative coherence and the full range of internal states for meaning-making, and extend the examination of mental health to other aspects, such as coping. Such analyses may provide additional insight into how parents of different cultures help children create meanings out of their emotionally negative experiences that can facilitate positive mental health.

Second, the present study examined emotional reminiscing in Chinese-American families and European-American families. Important within-group variations have been observed in both cultural communities in socialization practices (e.g., Wang et al., 1998; Wiley et al., 1998; Wang, 2013b). More research is called for to examine within-cultural and individual variations, especially in underrepresented populations, to understand the nuanced influences of culture. Furthermore, culture is transient and ever-changing, and future research needs to examine the impact of cultural change on family practices and associated developmental outcomes (Greenfield, 2018). For example, as a result of vast economic growth and Western influences in Mainland China in the past decades, native Chinese parents increasingly exhibit a parenting style that deviates from traditional practices (Wang and Hsueh, 2000; Chang et al., 2011; Lu and Chang, 2013). There are also increasing concerns for native Chinese children’s mental health (e.g., aggression and depression; e.g., Chen et al., 2012). Additional longitudinal research is required to identify effective parenting practices that facilitate children’s socioemotional functioning in the changing cultural context.

Third, given the long-term follow up, there was a 48% attrition rate and a relatively small final sample size. Analysis of the mother–child reminiscing variables revealed no difference between mother–child dyads who stayed in the study and those who dropped out. Still, future cross-cultural longitudinal studies with larger sample sizes will help to corroborate the present findings.

Lastly, the present study focused on middle-class European-American and Chinese-American families, with most of the parents being highly educated. Having the two cultural groups equivalent in socioeconomic status and parental education is in line with methodological conventions in cross-cultural research. Nonetheless, future research should be extended to different populations, such as working-class families (e.g., Wiley et al., 1998).

In conclusion, children’s daily experiences may not be all pleasant and positive. In face of emotionally negative events, how parents help children talk about and understand these experiences hold long-term implications for children’s mental health. Specific to helping children overcome their emotionally negative states and promoting long-term mental health, pertinent mother–child reminiscing characteristics are identified across two different cultural contexts. Culture also plays a role in shaping the link between parent–child reminiscing characteristics and child well-being.

## Data Availability Statement

The raw data supporting the conclusions of this article will be made available by the authors, without undue reservation.

## Ethics Statement

This study involving human participants was reviewed and approved by Institutional Review Board, Cornell University. Written informed consent to participate in this study was provided by the participants’ legal guardian/next of kin.

## Author Contributions

JBKK and QW contributed to the conceptualization and research design of the study. JBKK contributed to data collection and coding, performed data analysis, and took lead in drafting the manuscript. QW provided critical revisions. All authors approved the final version for submission.

## Funding

This research and publication were supported by NIH Grant R01-MH64661, NSF Award BSC-0721171 and a Hatch Grant from the U.S. Department of Agricultural to QW, and a Start-Up Grant from the Chinese University of Hong Kong, Shenzhen Presidential Fund PF01000977 to JBKK.

## Conflict of Interest

The authors declare that the research was conducted in the absence of any commercial or financial relationships that could be construed as a potential conflict of interest.

## Publisher’s Note

All claims expressed in this article are solely those of the authors and do not necessarily represent those of their affiliated organizations, or those of the publisher, the editors and the reviewers. Any product that may be evaluated in this article, or claim that may be made by its manufacturer, is not guaranteed or endorsed by the publisher.
